# Genome-wide identification, characterisation, and evolution of *ABF/AREB* subfamily in nine Rosaceae species and expression analysis in mei (*Prunus mume*)

**DOI:** 10.7717/peerj.10785

**Published:** 2021-02-04

**Authors:** Xue Yong, Tangchun Zheng, Xiaokang Zhuo, Sagheer Ahmad, Lulu Li, Ping Li, Jiayao Yu, Jia Wang, Tangren Cheng, Qixiang Zhang

**Affiliations:** 1 Beijing Advanced Innovation Center for Tree Breeding by Molecular Design, Beijing Forestry University, Beijing, China; 2 Beijing Key Laboratory of Ornamental Plants Germplasm Innovation & Molecular Breeding, Beijing Forestry University, Beijing, China; 3 National Engineering Research Center for Floriculture, Beijing Forestry University, Beijing, China; 4 Beijing Laboratory of Urban and Rural Ecological Environment, Beijing Forestry University, Beijing, China; 5 Engineering Research Center of Landscape Environment of Ministry of Education, Beijing Forestry University, Beijing, China; 6 Key Laboratory of Genetics and Breeding in Forest Trees and Ornamental Plants of Ministry of Education, Beijing Forestry University, Beijing, China

**Keywords:** Rosaceae, ABF/AREB, ABRE, Evolution, Expression, *Prunus mume*, Dormancy

## Abstract

Rosaceae is an important family containing some of the highly evolved fruit and ornamental plants. Abiotic stress responses play key roles in the seasonal growth and development of plants. However, the molecular basis of stress responses remains largely unknown in Rosaceae. Abscisic acid (ABA) is a stress hormone involving abiotic stress response pathways. The ABRE-binding factor/ABA-responsive element-binding protein (ABF/AREB) is a subfamily of the basic domain/leucine zipper (bZIP) transcription factor family. It plays an important role in the ABA-mediated signaling pathway. Here, we analyzed the *ABF/AREB* subfamily genes in nine Rosaceae species. A total of 64 *ABF/AREB* genes were identified, including 18, 28, and 18 genes in the Rosoideae, Amygdaloideae, and Maloideae traditional subfamilies, respectively. The evolutionary relationship of the *ABF/AREB* subfamily genes was studied through the phylogenetic analysis, the gene structure and conserved motif composition, Ka/Ks values, and interspecies colinearity. These gene sets were clustered into four groups. In the *Prunus ABF/AREB* (*PmABF*) promoters, several *cis*-elements related to light, hormone, and abiotic stress response were predicted. *PmABFs* expressed in five different tissues, except *PmABF5*, which expressed only in buds. In the dormancy stages,* PmABF1*, *2*, *5* and *7* showed differential expression. The expression of *PmABF3*, *4* and *6* was positively correlated with the ABA concentration. Except for *PmABF5*, all the *PmABFs* were sensitive to ABA. Several ABRE elements were contained in the promoters of *PmABF1*, *3*, *6*, *7*. Based on the findings of our study, we speculate that *PmABFs* may play a role in flower bud dormancy in *P. mume*.

## Introduction

Rosaceae is a large family spreading all over the world. It contains more than 3,000 species with various architectural forms. It is famous for its beautiful flowers and delicious fruits. Therefore, this family has significant ornamental, edible, and economic values. However, most of the Rosaceae species are distributed in the north temperate zone and face abiotic threats, such as low temperature and short photoperiod in winter. These stresses cause yield losses and physical damages to plants (Hirt, 2004).To cope with such threats, plants have evolved some strategies and the typical one is dormancy.

Abscisic acid plays a crucial role in the dormancy process (Chen et al., 2020b; Li et al., 2018; Song, Zhu & Yan, 2020; Tylewicz et al., 2018; Zheng et al., 2018). Environmental variations cause fluctuations of endogenous ABA levels in plants, resulting in multifarious physiological responses. The core ABA signaling pathway is mediated by the PYRABACTIN RESISTANCE/PYRABACTIN RESISTANCE-LIKE/regulatory component of the ABA receptor (PYR/PYL/RCAR receptors) coupled with PROTEIN PHOSPHATASE 2C (PP2C) and SNF1-RELATED PROTEIN KINASE 2 (SnRK2), with the downstream ABA-responsive bZIP transcription factors ABF/AREBs (Fujii et al., 2009; Fujita, Yoshida & Yamaguchi-Shinozaki, 2013).

It was found that ABA can regulate the expression events of thousands of genes (Matsui et al., 2008; Nemhauser, Hong & Chory, 2006). Most of the genes have a core *cis*-element named ABA-responsive element (ABRE; ACGTGG/TC) in their promoters (Zhang et al., 2005). Furthermore, a subfamily of basic leucine zipper (bZIP) transcription factor was involved in ABA regulation by binding to ABRE *cis*-element, so this subfamily was named ABRE-binding factors (ABFs) or ABRE-binding proteins (AREBs) (Choi et al., 2000; Fujita et al., 2005). Besides, the *Dc3* promoter-binding factors (DPBFs) were clustered into the ABF/AREB subfamily of the bZIP family because of their high homology (Kim et al., 2002). In the model plant *Arabidopsis*, nine members of the *ABF/AREB* subfamily were identified, including *AtABF1*, *AtABF2/AREB1*, *AtABF3*, *AtABF4/AREB2*, *AtDPBF1/ABI5*, *AtDPBF2*, *AtAREB3/DPBF3*, *AtDPBF4/EEL*, and *AtbZIP15* (Fujita et al., 2005; Kang et al., 2002; Kim et al., 2004; Kim, 2005; Kim et al., 2002; Yoshida et al., 2015; Yoshida et al., 2010).

Studies on the ABF/AREB TFs have been reported in several crop plants, such as rice (Miyazono et al., 2012), wheat (Rikiishi, Matsuura & Maekawa, 2010), soybean (Gao et al., 2011), potato (Liu et al., 2019b; Muniz García et al., 2012), sweetpotato (Wang et al., 2019), cotton (Kerr et al., 2018), tomato (Bastías et al., 2011; Bastías et al., 2014), Chinese cabbage (Bai et al., 2016), grape (Liu et al., 2019a; Zandkarimi et al., 2015), apple (Ma et al., 2017), strawberry (Li et al., 2016), and rose (Liu et al., 2017). However, the *ABF/AREB* subfamily remains poorly defined in Rosaceae. Therefore, a deep understanding of the *ABF/AREB* subfamily genes in Rosaceae can lay a theoretical foundation for improving their abiotic stresses adaptability.

So far, more than 14 Rosaceae species have been sequenced, and are available on the Genome Database for Rosaceae (GDR) (Jung et al., 2019). Here, nine Rosaceae species belonging to six major genera (*Fragaria*, *Malus*, *Prunus*, *Pyrus*, *Rosa*, and *Rubus*) and three traditional subfamilies (Rosoideae, Amygdaloideae, Maloideae) were selected to perform a comprehensive bioinformatic analysis (Folta & Gardiner, 2009). Moreover, we examined the promoter *cis*-elements of *ABF/AREB* genes in *P. mume* (*PmABFs*). Based on our previous transcriptome data, the expression patterns of *PmABFs* in different tissues and dormancy stages were analyzed. Besides, the relationship analysis between *PmABFs* gene expression and ABA content was performed during the dormancy process. Furthermore, we detected the response to exogenous ABA of *PmABFs*. The results of this study will be helpful to broaden the molecular biological functions of ABF/AREB TFs in the fruit and ornamental plants of Rosaceae.

## Materials & Methods

### Identification and phylogenetic analysis

In *Arabidopsis*, there are nine *ABF/AREB* subfamily genes: *AtABF1* (*At1g49720*), *AtABF2/AREB1* (*At1g45249*), *AtABF3* (*At4g34000*), *AtABF4/AREB2* (*At3g19290*), *AtABI5/ DPBF1* (*At2g36270*), *AtDPBF2* (*At3g44460*), *AtDPBF3/AREB3* (*At3g56850*), *AtDPBF4* (*At2g41070*), *AtbZIP15* (*At5g42910*) (Kim, 2005). The nine *Arabidopsis* ABF/AREB protein sequences, downloaded from tair (https://www.arabidopsis.org/), were used as queries to search the corresponding subject sequences in the genomic data of the nine Rosaceae species by Blastp with an *E*-value cut-off of 1e−22 to reduce false positives. The nine Rosaceae species are woodland strawberry (*Fragaria vesca*, *FvABFs*), rose (*Rosa Chinensis*, *RcABFs*), black raspberry (*Rubus occidentalis*, *RoABFs*), mei (*Prunus mume*, *PmABFs*), almond (*Prunus dulcis*, *PdABFs*), peach (*Prunus persica*, *PpABFs*), apricot (*Prunus armeniaca*, *ParABFs*), wild pear (*Pyrus betulifolia*, *PbABFs*) and apple (*Malus* × *domestica*, *MdABFs*). The genome files of mei and the eight Rosaceae species were downloaded from the NCBI (https://www.ncbi.nlm.nih.gov/genome/?term=13911) and GDB (https://www.rosaceae.org). The detailed genome information of the nine Rosaceae species was summarized in Table S1.

These protein sequences were further checked on Pfam (http://pfam.xfam.org/) and SMART (http://smart.embl-heidelberg.de/) database. These *ABF/AREB* subfamily genes were named in accordance with their location on the chromosome. The maximum likelihood (ML) phylogenetic tree was built by MEGA-X (Kumar et al., 2018) with full-length protein sequences aligned by Clustalx2.1, tested by 1,000 bootstrap replications, finally visualized by EvolView (http://www.evolgenius.info).

### Gene structure, motif prediction and protein characterization analysis

The gene structures of the *ABF/AREB* subfamily genes were predicted using GSDS 2.0 (http://gsds.gao-lab.org/). The conserved motifs of proteins were predicted on MEME v5.0.5 (http://meme-suite.org/tools/meme) (Bailey et al., 2015), with the following parameters: maximum number of motifs (20) and optimum motif width (6–50). The gene structures and motif distribution were plotted by TBtools (Chen et al., 2020a). The isoelectric point (pI), protein length, and molecular weight (MW) of these ABF/AREB subfamily protein sequences were calculated by the ExPasy ProtParam tool (https://web.expasy.org/protparam/).

### Physical localization, Ka (nonsynonymous)/Ks (synonymous) analysis

We got the information of the *ABF/AREB* subfamily genes on the corresponding chromosome according to the annotation documents and drew a sketch map of the gene physical location of each Rosaceae species through the MG2Cv2 website (http://mg2c.iask.in/mg2c_v2.0). The CDS sequences of the *ABF/AREB* subfamily genes were used to compute the Ka (non-synonymous rates) and Ks (synonymous rates) by DnaSP 6 software (Rozas et al., 2017). The Ka/Ks value was used to measure selection pressure. The divergence time was computed by the following formula: t = Ks/2*λ* ×10^−6^ Mya (in dicots, *λ* = 1.5 ×10^−8^) (Blanc & Wolfe, 2004).

### Promoter *cis*-element analysis and synteny analysis

The promoter sequences of *P. mume ABF/AREB* genes, 2 Kb upstream of the initiation codon (ATG), were extracted from the *P. mume* genome data. The promoter *cis*-element analysis was executed on the PlantCARE website (Lescot et al., 2002). The collinearity analysis between *P. mume* and the other eight Rosaceae species was performed by the Multiple Collinearity Scan toolkit (MCSscanX) (Wang et al., 2012). The visualization of the interspecific collinearity graphic was completed by TBtools (Chen et al., 2020a).

### Expression analysis of *ABF/AREB* in *P. mume* based on RNA-seq data

The RNA-seq data of five tissues (root, leaf, stem, fruit, bud) and dormancy stages of flower buds (EDI: November, EDII: December, EDIII: January, NF: February) used to analyze the expression patterns of *P. mume ABF/AREB* were obtained from our previously published research (Zhang et al., 2012; Zhang et al., 2018). The heat maps were generated by TBtools (Chen et al., 2020a).

### ABA treatment of plant material

The plant material used in this study was *P. mume* cv. ‘Lve’, which was cultivated in the nursery of Beijing Forestry University (40°07′N, 116°11′E). To test the effect of ABA on *PmABFs*, the new branches with flower buds were cut off and sprayed with 100 mg/L ABA. After 0, 2, 4, 8, 12, 24, 48, and 72 h, flower buds were harvested in liquid nitrogen and stored at −80 °C for RNA extraction.

### RNA extraction and qRT-PCR analysis of *ABF/AREB* in *P. mume*

The total RNA was isolated by EASYspin Plus Plant RNA Kit (Aidlab, Beijing, China), and the first-strand cDNA was synthesized by TIANScript First Strand cDNA Synthesis Kit (Tiangen, Beijing, China). The qRT-PCR was performed on the PikoReal real-time PCR system (Thermo Fisher Scientific, CA, USA) with a 10 µL reaction volume, including 5 µL of SYBR Premix ExTaq II (Takara, Dalian, China), 1  µL of cDNA, and 0.2 µL of each primer (Table S3). The reactions were carried out under the following conditions: 95 °C for 30 s, 40 cycles of 95 °C for 5 s and 60 °C for 30 s, and finally end in 20 °C. The *protein phosphatase 2A* (*PP2A*) gene of *P. mume* was selected to be the reference gene for normalization (Wang et al., 2014). The 2^−ΔΔ*Ct*^ method was used to calculate the relative expression level, and each experiment was repeated in triplicate.

## Results

### Identification, characteristics and phylogenetic analysis of *ABF/AREBs* in Rosaceae

To identify the *ABF/AREB* subfamily genes in nine Rosaceae species (woodland strawberry, rose, black raspberry, mei, almond, peach, sweet cherry, apricot, wild pear, and apple), Blastp searches were executed against each species’ genome data with an *E*-value of 1e−22. All of the nine species are diploid except the triploid apple. In this study, a total of 64 highly conserved *ABF/AREB* subfamily genes were identified. The protein sequence data and sequence information are shown in Data S1 and Table S2. The species in the same subfamily has the same number of *ABF/AREB* genes, as shown in Table 1, there were six, seven, and nine *ABF/AREB* genes in Rosoideae species (*F. vesca*, *R. occidentalis*, and *R. Chinensis*), *Prunus* species (*P. mume*, *P*. *armeniaca*, *P. dulcis*, and *P. persica*) and Maloideae species (*P. betulifolia* and *M.* × *domestica*), respectively (Table 1). The characteristics of ABF/AREB protein sequences were calculated by the ExPASy ProtParam server. As shown in Table S2, the sequence length ranges from 260 aa (PavABF5) to 1,096 aa (MdABF7), the molecular weight ranges from 29.07 KDa (PavABF5) to 122 KDa (MdABF7), and the isoelectric point (pI) ranges from 4.29 (ParABF4) to 9.82 (RoABF2). In *P. mume*, the shortest and the longest protein sequences were PmABF2 (264 aa) and PmABF7 (614 aa), respectively. The isoelectric point (pI) of PmABFs varied from 4.96 (PmABF1) to 9.68 (PmABF3). The gene name, corresponding gene ID, and locus are shown in Table S2.

**Table 1:** *ABF/AREB* genes number and their chromosomal distribution of nine Rosaceae species.

Troditional subfamily	Genus name	Species name	Chromosome number	Identified *ABF/AREB* genes	Chromosomal distribution of *ABF/AREB* genes
Rosoideae	Fragaria	*Fragaria vesca*	*x* = 7	6	Chr.2,3,5,6,7
	Rubus	*Rubus occidentalis*			
	Rosa	*Rosa Chinensis* ‘Old Blush’			Chr.1,3,5,6
Amygdaloideae	Prunus	*Prunus mume*	*x* = 8	7	Chr.1,2,5,6,8
		*Prunus armeniaca*			
		*Prunus dulcis* ‘Texas’			Chr.1,2,6,7,8
		*Prunus persica*			
Maloideae	Pyrus	*Pyrus betulifolia*	*x* = 17	9	Chr.2,3,5,7,8,12,14,15
	Malus	*Malus domestica* ‘HF’			

**Figure 1:**
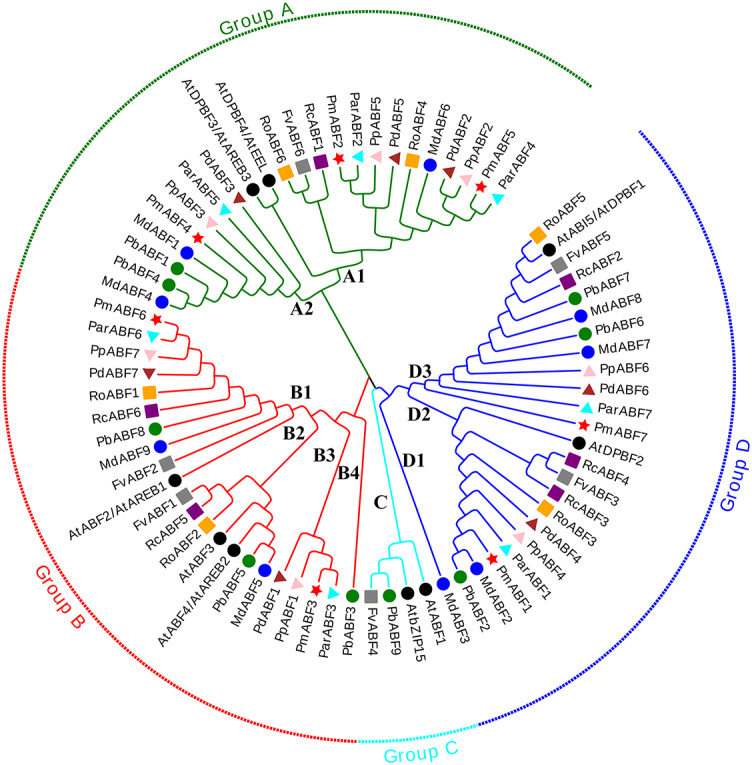
Phylogenetic tree analysis of *ABF/AREB* genes from nine Rosaceae species and *Arabidopsis* by MEGA. The Phylogenetic tree was divided into four groups, which are represented by green, red, cyan and blue branch lines. At, *A. thaliana*; Pm (*P. mume*); Pd (*P. dulcis*)*;* Pp (*P. persica*); Par (*P. armeniaca*); Md (*M.* × *domestica*); Pb (*P. betulifolia*); Rc (*R. chinensis*); Ro (*R. occidentalis*); Fv (*F. vesca*). Different species are marked with different colors and shapes, *PmABFs* are labeled with red stars.

The phylogenetic tree was constructed by the maximum-likelihood (ML) method using MEGA_X with 73 ABF/AREB protein sequences, consisting of 9 *Arabidopsis* sequences and 64 sequences from nine Rosaceae species. These *ABF/AREB* subfamily genes were divided into four groups (A, B, C, and D; Fig. 1). Group C was the smallest group, containing only two Rosaceae *ABF/AREB* genes: one woodland strawberry gene (*FvABF4*) and one wild pear gene (*PbABF9*). The other 69 *ABF/AREB* subfamily genes were distributed evenly in Group A (23 genes), B (22 genes), and D (24 genes). Each of the three groups (A, B, and D) contained all nine Rosaceae species. Group A was divided into two subgroups: A1 (15 genes) and A2 (8 genes). Group B was divided into four subgroups (B1-B4). Subgroup B1 and B2 clustered first and then clustered with Subgroup B3 and B4. Group D was divided into three subgroups (D1-D3). Subgroup D2 and D3 were grouped first and then clustered with Subgroup D1. As summarized in Table 2, most Rosaceae species in group A had three *ABF/AREB* genes. However, *P. betulifolia* and *R. occidentalis* had two genes, and *F. vesca* and *R. Chinensis* had one gene. In Group B, all nine Rosaceae species possessed two *ABF/AREB* genes except *P. betulifolia* (3 genes). In Group C, only *F. vesca* and *P. betulifolia* had one *ABF/AREB* gene. In Group D, six Rosaceae species contained two *ABF/AREB* genes, however, *P. betulifolia* and *R. Chinensis* contained three, and *M.* × *domestica* contained four.

### Gene structure and conserved motif distribution of *ABF/AREBs* in Rosaceae

**Figure 2:**
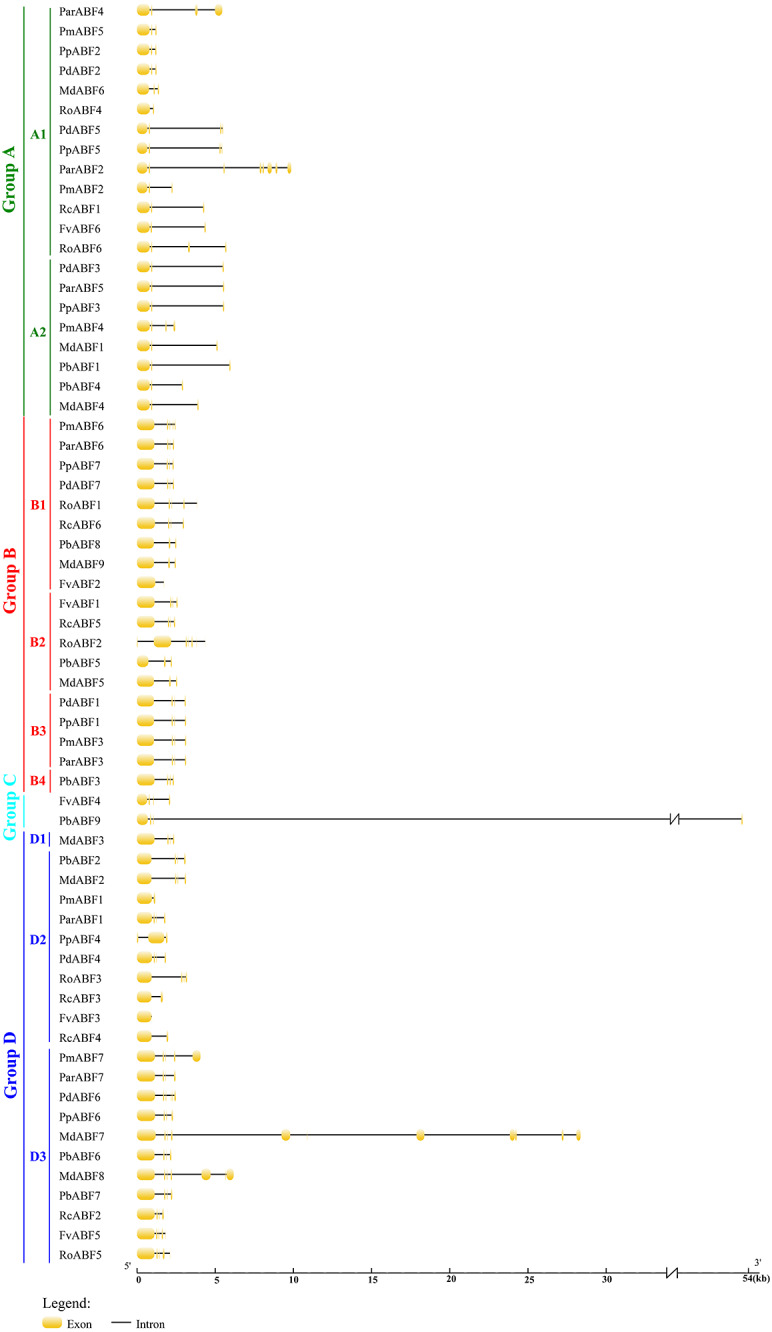
Gene structure of *ABF/AREB* genes in Rosaceae using the GSDS program. The orange round-corner rectangle represents exons, and the black line represents intron.

To better understand the relationships of the ABF/AREB proteins, the gene structures were constructed by GSDS2.0. As shown in Fig. 2, the structure of *ABF/AREB* was usually similar in each subgroup. However, there were few exceptions, such as *PbABF9* (Group C) had the longest sequence, followed by the *MdABF7* (Subgroup D3) and *ParABF2* (Subgroup A1), and the *FvABF3* (Subgroup D2) had the shortest sequence. Also, *MdABF7* and *ParABF2* contained more exons and introns. The location of exons and introns in *RoABF2* (Subgroup B2) and *PpABF4* (Subgroup D2) was different in *ABF/AREB* subfamily. Most of the *ABF/AREB* genes in nine Rosaceae species contained three or four exons. In Group A, 66.7% had three exons and 23.8% had four exons. In Group B, 26.3% had three exons and 57.9% had four exons. In Group C, the two sequences had four exons. In Group D, half of the members had four exons. This suggests that the evolution of these genes was conservative.

**Table 2:** The number of genes identified at different groups of the *ABF/AREB* family.

Subfamily	Species	GroupA	GroupB	GroupC	GroupD	Total
	*Arabidopsis thaliana*	2	3	2	2	9
Rosoideae	*Fragaria vesca*	1	2	1	2	6
	*Rosa Chinensis* ‘Old Blush’	1	2	0	3	6
	*Rubus occidentalis*	2	2	0	2	6
Amygdaloideae	*Prunus mume*	3	2	0	2	7
	*Prunus dulcis* ‘Texas’	3	2	0	2	7
	*Prunus persica*	3	2	0	2	7
	*Prunus armeniaca*	3	2	0	2	7
Maloideae	*Pyrus betulifolia*	2	3	1	3	9
	*Malus domestica* ‘HF’	3	2	0	4	9
Total		23	22	4	24	73

**Figure 3:**
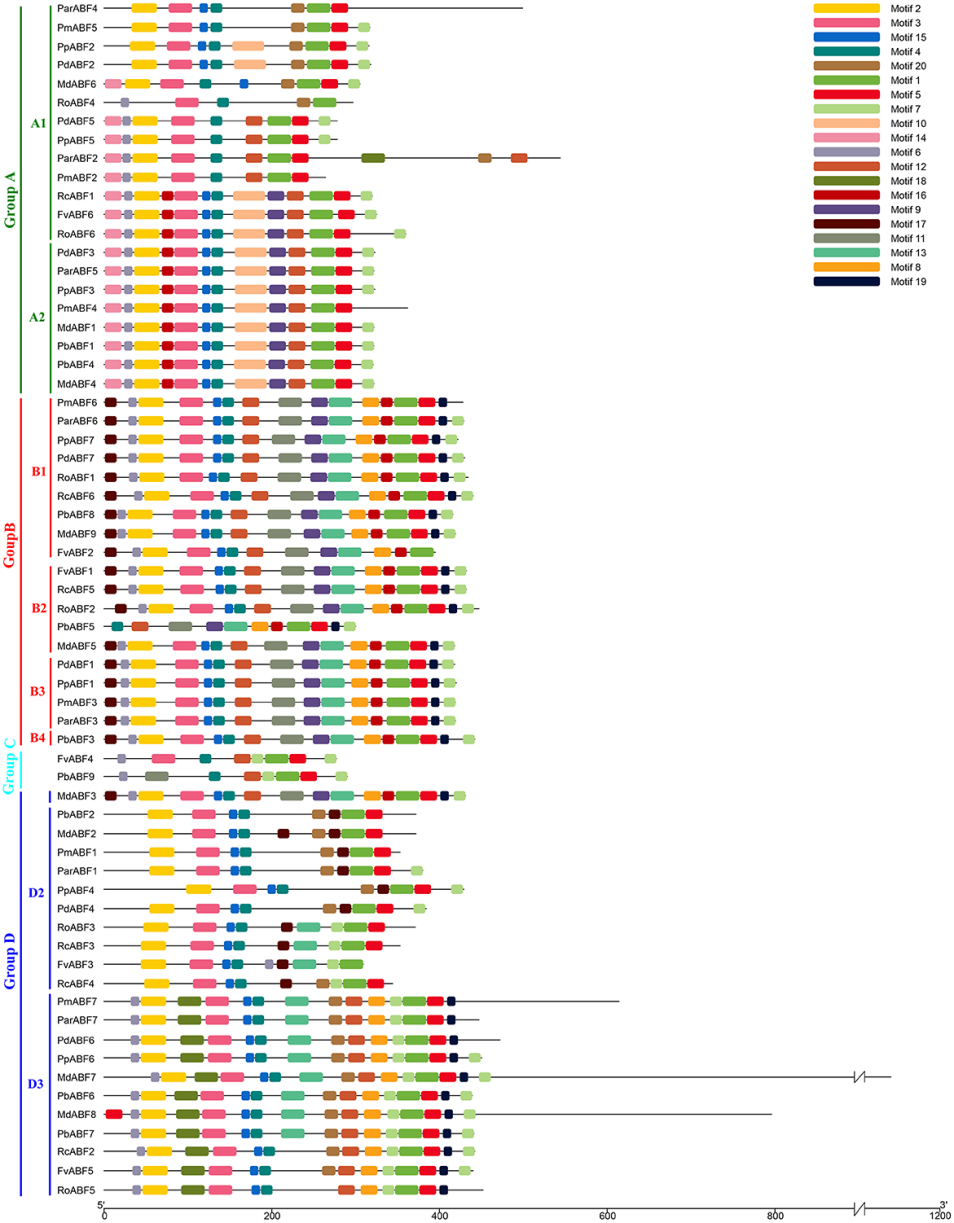
Conserved motifs distribution of *ABF/AREBs* in Rosaceae identified by MEME. These motifs logos were shown in Fig. S1.

Twenty conserved motifs (Fig. S1) in the ABF/AREB protein sequences of nine Rosaceae species were detected by MEME. Most of the sequences were clustered in the same subfamily and had similar motifs with similar sizes in similar places. As shown in Fig. 3, the most conserved Motif 1 was found in all the 64 ABF/AREB protein members. Motif 2, motif 3, motif 4, motif 5, motif 7, motif 12 and motif 15 were found in the majority of ABF/AREB protein sequences, and with an occurrence percentage of 93.8%, 96.9%, 98.4%, 95.3%, 84.4%, 75% and 89.1%, respectively. Motif 10 and motif 14 were only found in Group A, motif 11 was found in Group B and C. Besides, motif 9 and motif 16 coexisted in half of the group A members, and all the Group B and Subgroup D1 members. Furthermore, motif 8, motif 13, motif 17, and motif 19 were specifically included in Group B and D.

**Figure 4:**
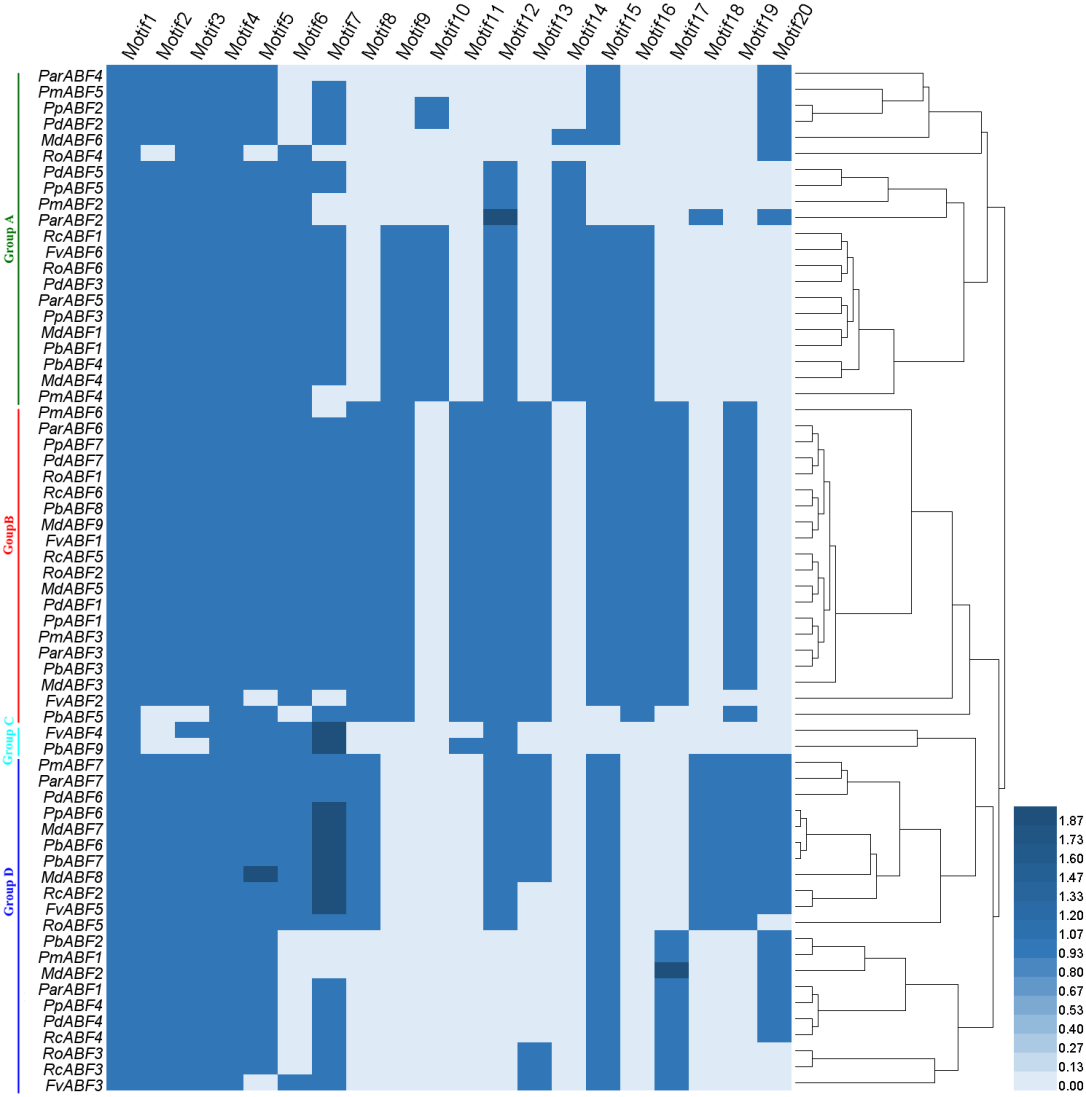
Heatmap with a phylogenetic tree of the conserved motifs composition of *ABF/AREBs* in Rosaceae generated by the type and number of motifs.

Based on the distribution of the conserved motifs, a heatmap containing a phylogenetic tree was generated based on the type and number of motifs (Fig. 4). In this phylogenetic tree, genes with the same types and numbers of motifs are always preferentially clustered together. Comparing it with the phylogenetic analysis result (Fig. 1), we found that the group classification results by two methods were mainly the same but with some differences in detail. In Group A, the members in the subgroups were different from those in the phylogenetic analysis result (Fig. 1), some genes (*RcABF1*, *FvABF6*, and *RoABF6*) in Subgroup A1 of Fig. 1 migrated with Subgroup A2 because of their common types and numbers of motifs. In Group B, most genes had the same types and numbers of motifs clustered first, the one lack of one motif type (*PmABF6*) clustered subsequently, the two genes lack several motifs (*FvABF2* and *PbABF5*) were classed into the group finally. There was no change in Group C. In Group D, only the Subgroup D1 member *MdABF3* of Fig. 1 moved to Group B in the heatmap of Fig. 4, the other subgroup members did not change.

### Chromosomal location and Ka/Ks ratio of *ABF/AREBs* in Rosaceae

The chromosomal location maps of the 64 *ABF/AREB* subfamily genes were constructed by MG2Cv2.1 (Fig. S2). As shown in Fig. S2 and Table 1, the chromosomal distribution of *ABF/AREB* genes was the same in *F. vesca* and *R. occidentalis*. In the four *Prunus* plants, the distribution of *PmABFs* and *ParABFs*, *PdABFs* and *PpABFs* were identical on chromosomes, respectively. The chromosomal location of *ABF/AREBs* was the same in *P. betulifolia* and *M.* × *domestica*.

To explore the selection pressure in the evolution of *ABF/AREB* subfamily genes, the Ka/Ks values were calculated for nine Rosaceae species (Table S4). The Ka/Ks ratio is the basis to estimate selection pressure. A Ka/Ks value of less than one implies purifying selection, Ka/Ks = 1 represents neutral selection and Ka/Ks >1 indicates positive selection (Li et al., 2009). In total, 95 *ABF/AREB* subfamily gene paralogs were found in the nine Rosaceae species. The Ka/Ks values of all these gene paralogs in woodland strawberry (7), rose (6), mei (8), almond (9), peach (8), wild pear (24) and apple (17) were less than one, suggesting that these genes evolved under purifying selection. In black raspberry (8) and apricot (8), most of the Ka/Ks values were less than one except three gene paralogs (*RoABF5*/*RoABF1*, *RoABF5*/*RoABF2*, and *ParABF2*/*ParABF5*). The Ka/Ks values of the three gene paralogs were greater than one, which indicates that they were evolved under positive selection.

The divergence time was calculated based on Ks values. The divergence time of 57.9% of the 95 gene paralogs was more than 50 Mya. There were 7 gene paralogs with divergence time over 100 Mya, including *PdABF2*/*PdABF5*, *PdABF3*/*PdABF5*, *PpABF3*/*PpABF5*, *PbABF6*/*PbABF9*, *PbABF6*/*PbABF3*, *PbABF9*/*PbABF4*, *FvABF5*/*FvABF1* (Table S4). This shows that these gene paralogs are relatively ancient.

### Collinearity relationship of *ABF/AREBs* in Rosaceae

**Figure 5:**
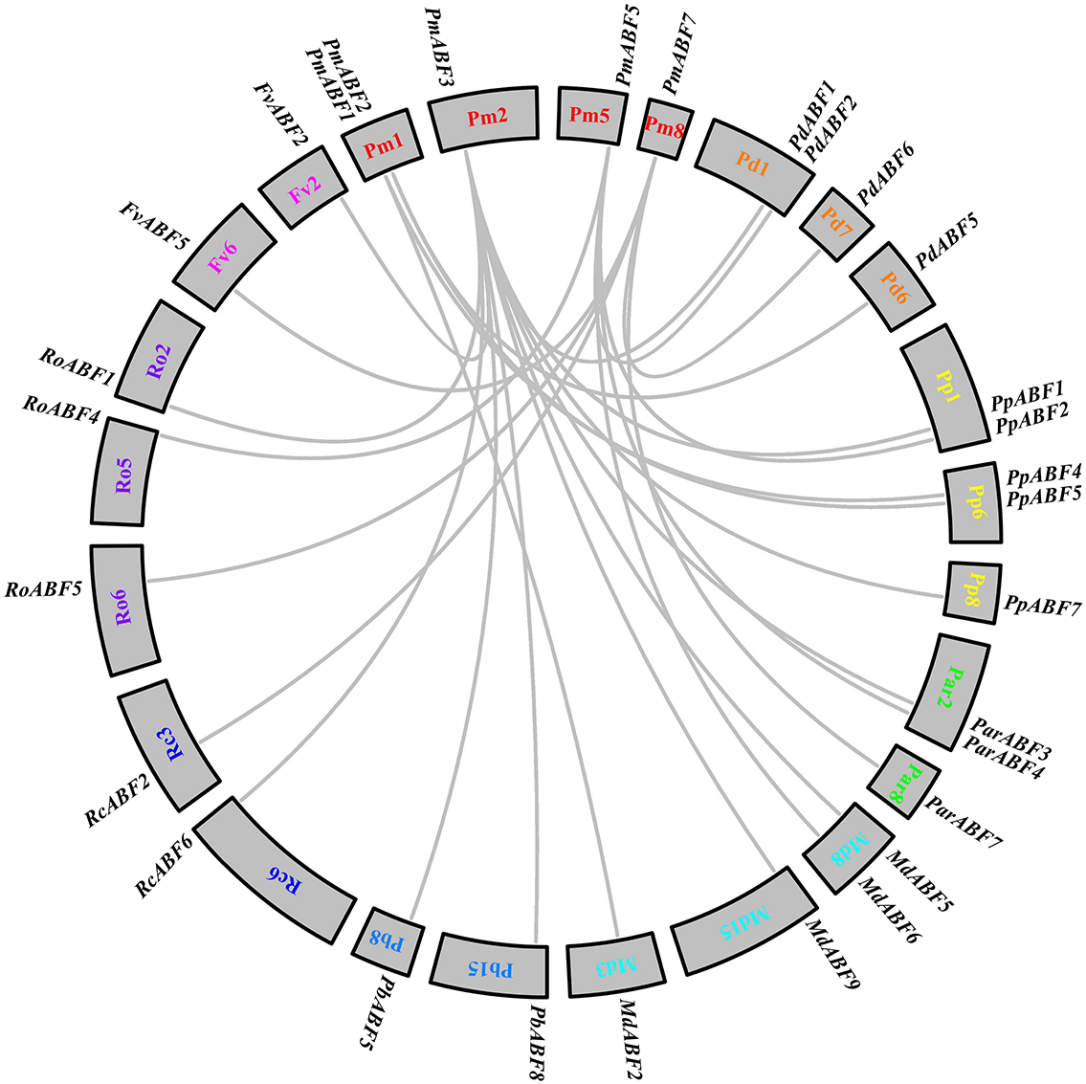
Interspecific collinearity analysis of the *ABF/AREB* genes. Different species were labelled by different chromosome numbers with different colors: Pm (*P. mume*); Pd (*P. dulcis*)*;* Pp (*P. persica*); Par (*P. armeniaca*); Md (*M.* × *domestica*); Pb (*P. betulifolia*) ; Rc (*R. chinensis*); Ro (*R. occidentalis*); Fv (*F. vesca*).The gray lines represent the syntenic gene pairs.

To explore the evolutionary relationships between *P. mume* and other eight Rosaceae species, collinearity analysis was performed by MCScan. A total of 25 collinear gene pairs were identified between them. As shown in Fig. 5, two orthologous gene pairs were found between Pm (*P. mume*) and Pb (*P. betulifolia*), Rc (*R. chinensis*) and Fv (*F. vesca*), respectively. There were three orthologous gene pairs between Pm (*P. mume*) and Par (*P. armeniaca*) or Ro (*R. occidentalis*). Between Pm (*P. mume*) and Pd (*P. dulcis*) or Md (*M.* × *domestica*), four orthologous gene pairs were identified. And, the number of orthologous gene pairs between Pm (*P. mume*) and Pp (*P. persica*) was five. Two genes were homologous to *PmABF1* or *PmABF2*. Four and five orthologous gene pairs were orthologous to *PmABF5* and *PmABF7*, respectively. Ten genes were orthologous to *PmABF3*, which showed a collinear relationship with two *ABF* genes from each of *P. persica* (*PpABF1* and *PpABF7*), *M.* × *domestica* (*MdABF5* and *MdABF9*) or *P. betulifolia* (*PbABF5* and *PbABF8*). The results of the collinearity analysis of the *ABF/AREB* subfamily genes were consistent with the phylogenetic analysis (Fig. 1).

### Promoter *cis*-elements composition of *PmABFs*

**Figure 6:**
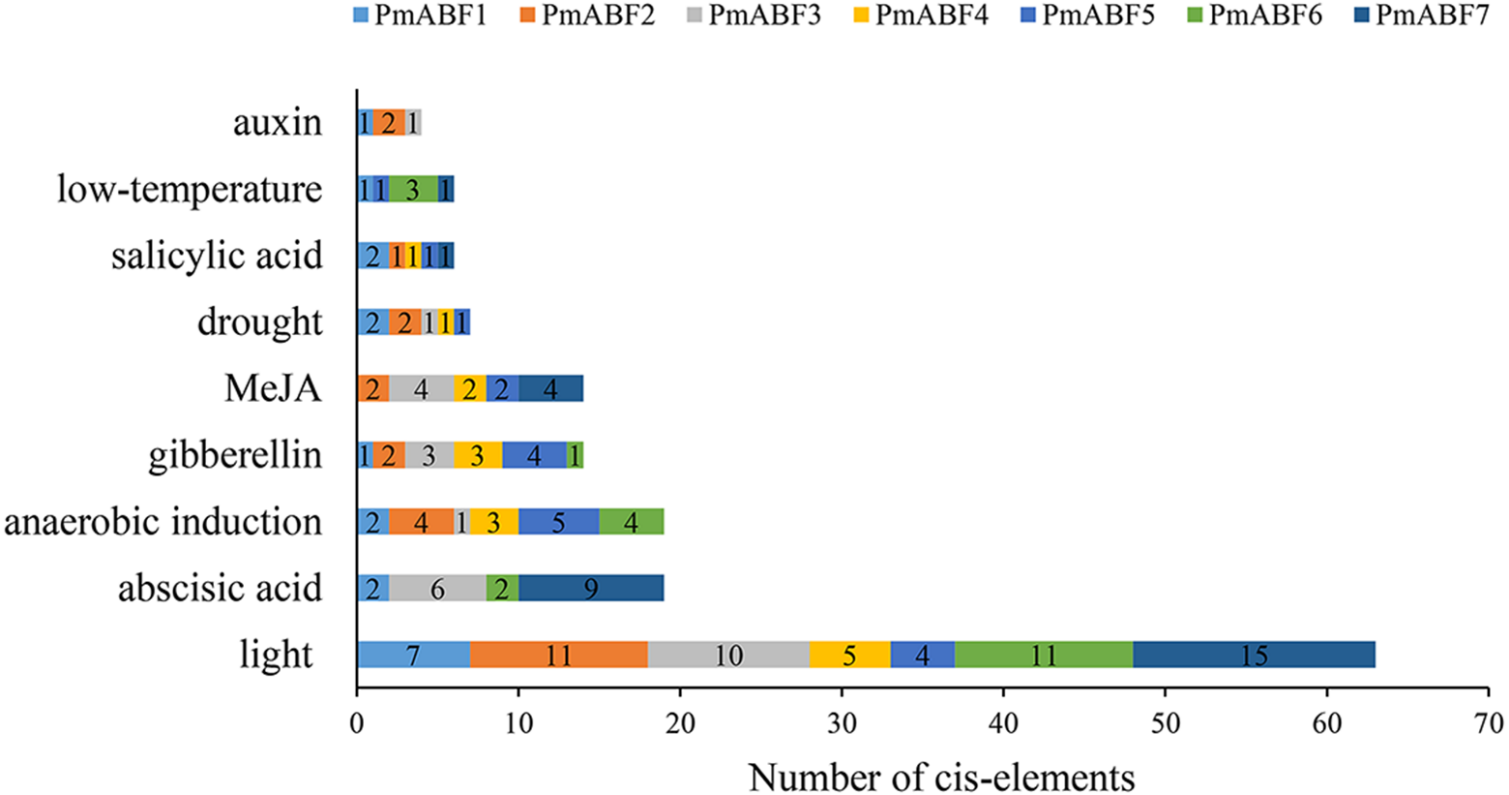
Promoter *cis*-elements of *PmABFs*. The seven *PmABFs* genes are represented by different colors.

The promoter *cis*-elements can regulate the gene expression level. Here, we analyzed the *cis*-elements composition of seven *PmABFs* promoters with the length of 2 Kb (Data S2) using PlantCARE. Three main types of *cis*-acting elements were identified, including light, hormone, and stress response elements. Each promoter of the seven *PmABFs* had 4–15 light-response elements (Fig. 6, Table S5), such as GT1-motif, G-box, Box 4, Gap-box, AE-box, and MRE. Hormone-response regulatory elements consisted of 19 abscisic acid (ABA) response elements (ABRE), 14 gibberellin (GA) responsive elements (TATC-box, P-box, GARE-motif), 14 methyl jasmonate (MeJA) response elements (TGACG-motif and CGTCA-motif), 6 salicylic acid (SA) related TCA-elements and 4 auxin (IAA) related TGA-elements. The ABA/ GA/ MeJA/ SA/ IAA-response elements were found in the promoters of *PmABF1*, *3*, *6*, *7*, *PmABF1-6*, *PmABF2-5*, *7*, *PmABF1*, *2*, *4*, *5*, *7*, and *PmABF1-3*, respectively. The abscisic acid response ABRE element was found in four *PmABF* members (*PmABF1*, *3*, *6*, and *7*). *PmABF7* contained the most abundant ABRE elements (9) among the four genes. Moreover, the stress response related elements contained 19 anaerobic induction elements (ARE), 7 drought induction related elements (MBS), and 5 low-temperature responsive elements (LTR). These stress response elements were related to anaerobic induction (all *PmABFs* except *PmABF7*), drought (all *PmABFs* except for *PmABF6* and *PmABF7*), and low temperature (*PmABF1* and *PmABF7*). The detailed information of the promoter *cis*-elements composition in *PmABFs* is shown in Table S5.

### Expression patterns of *PmABF* in different tissues and dormancy stages of flower buds

**Figure 7:**
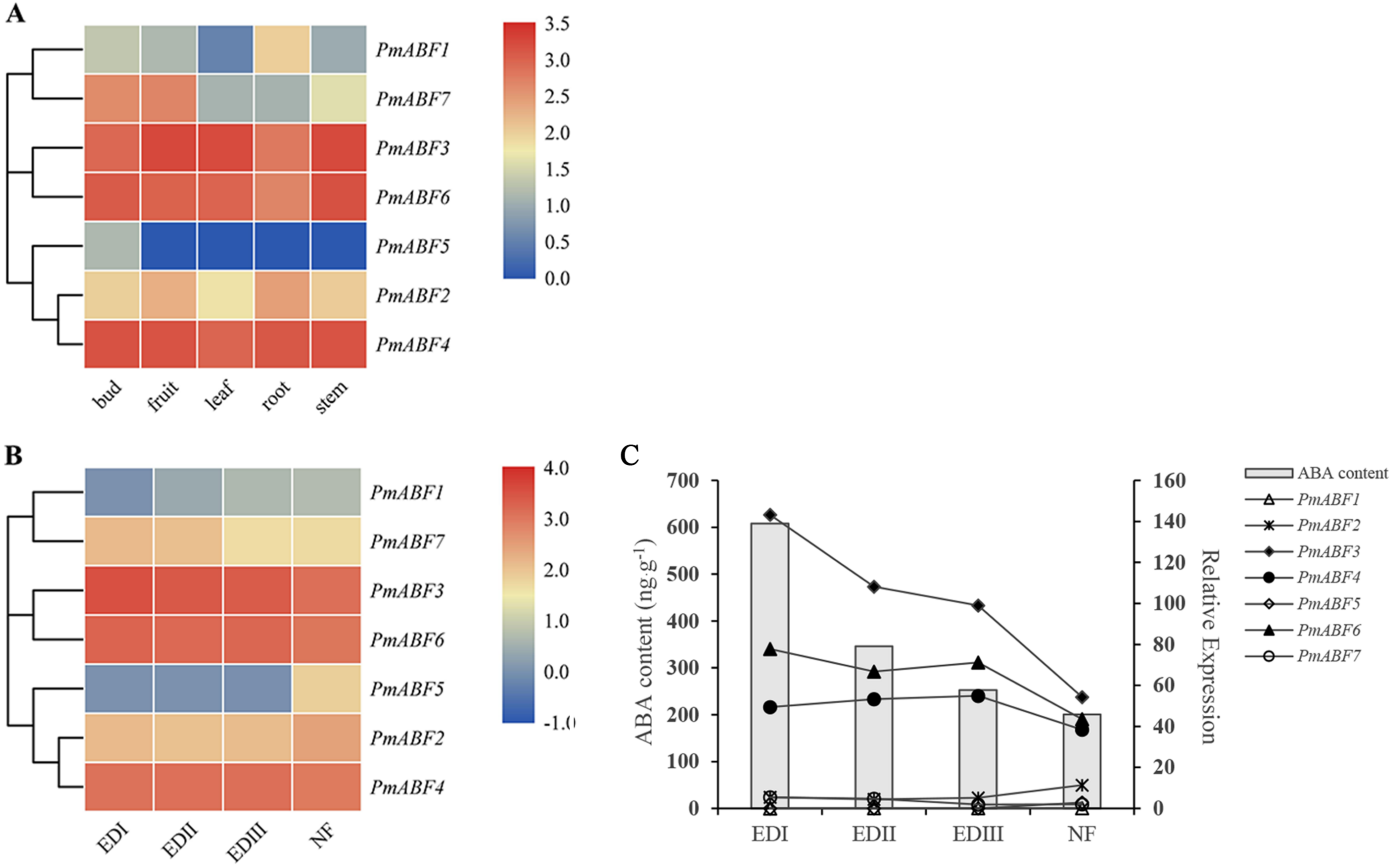
(A) Tissue-specific expression patterns of *PmABFs*. (B) Expression patterns of *PmABFs* in flower bud during dormancy stages. (C) The relationship between the expression of *PmABFs* and ABA content in flower bud during dormancy stages. EDI: November, EDII: December, EDIII: January, NF: February.

For further understanding of the function of *PmABFs*, the expression patterns of *PmABFs* in five different tissues (bud, fruit, leaf, root, and stem) and four dormancy stages of flower buds (EDI in November; EDII in December; EDIII in January and NF in February) were analyzed based on our previous transcriptome data (Zhang et al., 2012; Zhang et al., 2018). Except for *PmABF1*, the remaining six genes expressed in all five tissues (Fig. 7A). *PmABF3*, *4* and *6* showed high expression in each tissue type. *PmABF1* showed low expression in all tissues except the root. *PmABF7* showed high expression in bud and fruit. *PmABF5* was specifically expressed in the bud at a very low level. During the EDI-III stages, the flower buds remained dormant and released in the NF stage. As shown in Fig. 7B, the expression level of *PmABF3, 4* and *6* was high and stable in the dormancy stages. *PmABF1* was not expressed in EDI and maintained a very low expression in the next three periods. Compared to *PmABF3*, *4* and *6*, the expression levels of *PmABF2* and *PmABF7* were comparatively low with slight fluctuation in the dormancy periods. The expression of *PmABF2* was stable from EDI to EDIII and increased in NF. The expression of *PmABF7* was similar in EDI and EDII, EDIII and NF, but the level decreased in EDIII and NF. *PmABF5* showed no expression in EDI- EDIII, however, its expression was suddenly increased in NF.

### Expression patterns of *PmABF* in response to ABA

**Figure 8:**
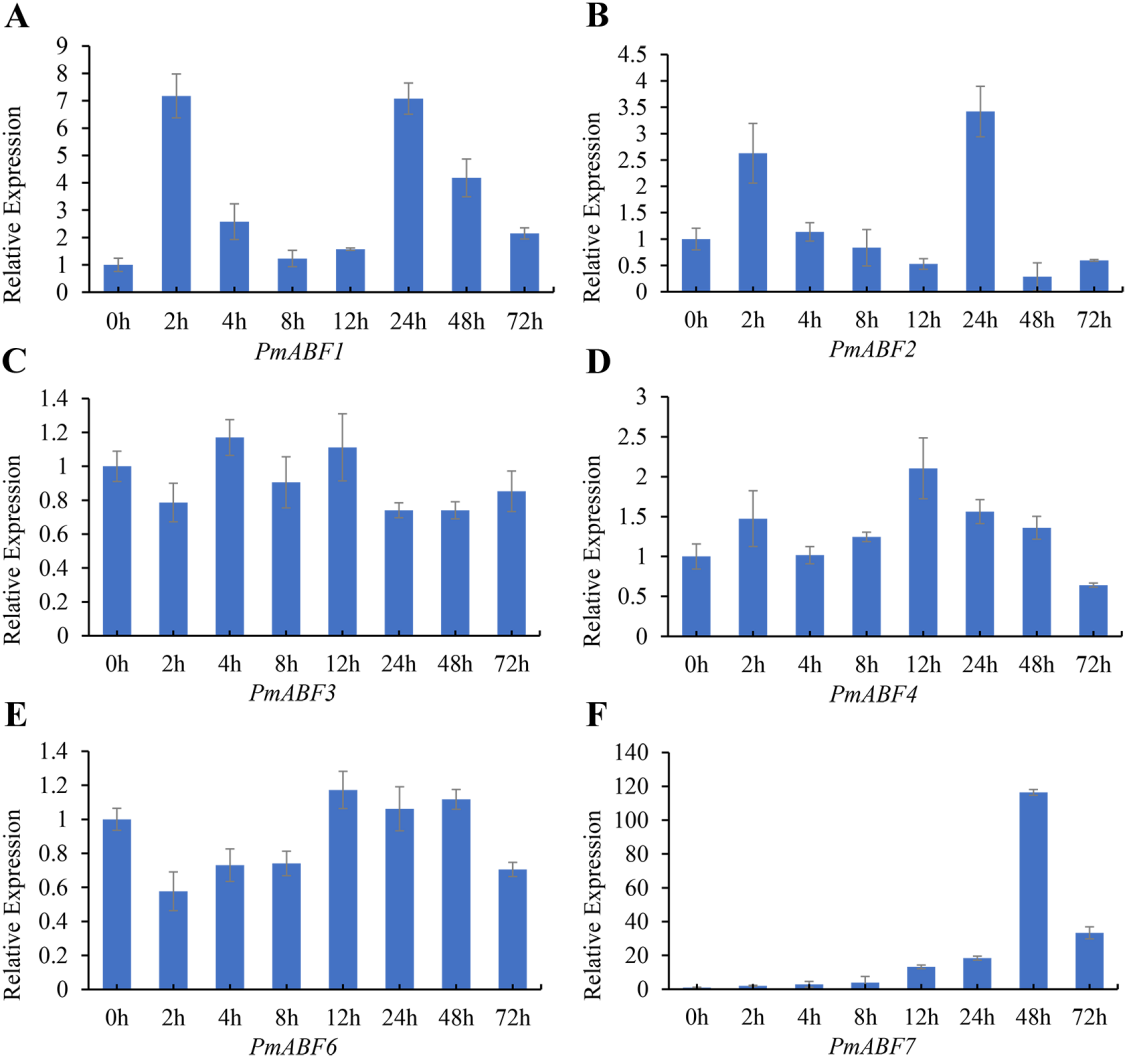
Expression patterns of *PmABFs* under ABA treatment. (A) PmABF1, (B) PmABF1, (C) PmABF3 (D) PmABF4, (E) PmABF6, (F) PmABF7.

It has been reported that ABA can induce *ABF/AREB* subfamily genes in *Arabidopsis* (Choi et al., 2000; Fujita et al., 2005; Uno et al., 2000; Yoshida et al., 2015). To evaluate the ABA-responsive expression of *PmABFs* genes, flower buds were treated with 100 mg/L ABA solution, and the qRT-PCR analyses were performed. The results of ABA-responsive expression pattern analysis are shown in Fig. 8. The expression level of all the *PmABFs* increased at different time points after ABA treatment. However, the expression of *PmABF5* was negligible. *PmABF1*, *PmABF2* and *PmABF7* showed significant expression after ABA stress. The expression of *PmABF1* and *PmABF2* was obviously induced after 2 h, decreased rapidly from 4–12 h, and then suddenly increased after 24 h, finally dropped to a low level. The expression of *PmABF7* was gradually induced, peaked at 48 h, and then decreased at 72 h. However, the expression level of *PmABF* 3 fluctuated slightly under the ABA treatment, the transcripts of *PmABF4* and *PmABF6* were accumulated at 12 h (Fig. 8). Therefore, the *P. mume ABF/AREB* subfamily genes showed an obvious response to ABA, but with different intensities.

## Discussion

Abiotic stress is a great risk to the growth and development of plants. Several transcription factors have been involved in plant abiotic stress responses (Yoon et al., 2020). ABF/AREB transcription factor is one of the important transcription factors responsible for abiotic stress responses. It participates in ABA signal transduction and stress response in plants (Choi et al., 2000; Corrêa et al., 2008; Kim, 2005). In this study, 64 members of the *ABF/AREB* subfamily in nine Rosaceae species were identified (Fig. 1 and Table 1). The species belong to three traditional subfamilies of Rosaceae, including Rosoideae, Amygdaloideae, and Maloideae. The *ABF/AREBs* genes in the same subfamily usually clustered together. The phylogenetic analysis result was accorded with the taxonomy result.

The chromosome number of Rosoideae, Amygdaloideae, and Maloideae were *x* = 7, 8, 17 with the *ABF/AREB* genes number *n* = 6, 7, 9, respectively. The number of *ABF/AREB* s varied in different subfamilies, the more chromosomes the species have, the more *ABF/AREB* genes there are. The Maloideae was considered to be the origin of other subfamilies in Rosaceae, because of the most chromosome number (*x* = 17) (Folta & Gardiner, 2009). The chromosome distribution of *ABF/AREBs* in each traditional subfamily is partially similar. This may be due to the plant whole-genome duplication events (WGD), gene tandem duplications and chromosome recombination. Regarding the Ka/Ks value (Table S4), 95 *ABF/AREB* subfamily gene paralogs were counted. Only three gene paralogs (*RoABF5*/*RoABF1*, *RoABF5*/*RoABF2*, and *ParABF2*/*ParABF5*) possessed a Ka/Ks value greater than one, suggesting their evolution under positive selection. Therefore, they would be remained in the future evolution.

The 64 *ABF/AREB* genes were divided into 4 groups, based on the phylogenetic analysis (Fig. 1). Most of these genes were evenly distributed in Group A, B and D, except for *FvABF4* and *PbABF9* (in Group C). These groups consist of several subgroups, in each subgroup, a majority of genes showed a similar structure, including gene length, exon, and intron number (Fig. 2). Moreover, the conserved motif constructions of *ABF/AREB* genes in different subgroups were identical in part with similar size and position (Fig. 3 and Fig. 4). Based on the conserved motif constructions, a heatmap with a phylogenetic tree (Fig. 4) was built. The conserved motif classification results supported the previous phylogenetic analysis result (Fig. 1), showing the reliability of the previous phylogenetic analysis. In every subgroup, the gene structure and conserved motif construction were relatively conserved, suggesting that their members might have similar gene functions. However, the gene structure and conserved motif constructions of the two genes (*FvABF4* and *PbABF9*) from group C were different from others. Perhaps, they had some special function.

As previously established, gene expression was affected by *cis*-elements of the promoter. Three major types of *cis*-acting elements (light, hormone, and stress response elements) were distributed in *PmABF* gene promoters (Fig. 6 and Table S5). It is illustrated that the expression of *PmABFs* may be induced by light, hormones (ABA, GA, MeJA, SA, and IAA), and some abiotic stresses (oxygen deficit, drought, and low temperature). This is consistent with previous studies. In *Arabidopsis*, *AtABFs* were induced by light, ABA, and stress (drought, salt, low temperature) (Choi et al., 2000; Kim et al., 2004; Xu et al., 2014). *StABF1*, in potato, can be induced by ABA, drought, cold, and salt stress (Muniz García et al., 2012). Four *VvABF/AREBs* were regulated by drought and salt stress at the transcriptional level (Zandkarimi et al., 2015). However, *PmABFs* can be induced by not only light, ABA, drought, and low-temperature stress but also by other hormones (GA, MeJA, SA, and IAA), and oxygen deficit stress.

Gene expression analysis is the basis of gene function exploration. *PmABF3*, *4* and *6* showed high expression in all tissues, but the other four genes (*PmABF1*, *2*, *5*, and *7*) showed relatively low expression in specific tissues (Fig. 7A). *PmABF1* and *PmABF5* expressed mainly in roots and flower buds. In the dormancy stages, *PmABF3*, *4* and *6* also showed high expression, while *PmABF1*, *2*, *5* and *7* expressed differentially. In our previous study, the content of ABA during dormancy stages was measured because of its important role in dormancy (Zhang et al., 2018). From EDI to NF stages, the content of ABA decreased gradually. In order to understand the relationship between the expression of *PmABFs* and ABA content during dormancy stages, a correlation map was plotted. As shown in Fig. 7C, the expression level of *PmABF3*, *PmABF4* and *PmABF6* decreased with the decrease of ABA content.

It has been reported that *ABF/AREBs* subfamily genes were responsive to ABA in several species (Choi et al., 2000; Liu et al., 2019b; Wang et al., 2019; Yang et al., 2020). In this study, except for *PmABF5*, the other six *PmABFs* responded to ABA with different expression patterns. The expression of these six genes was induced at different time points after the exogenous treatment of ABA (Fig. 8). Among them, *PmABF1*, *2* and *7* were very sensitive to ABA, in particular *PmABF7*, which showed significantly high expression after 48 h of ABA treatment (Fig. 8). However, *PmABF3*, *4* and *6* showed a mild response to ABA. According to the promoter *cis*-acting elements analysis, there were two, six, two and nine ABRE elements that were found in the 2 Kb promoter of *PmABF1*, *3*, *6* and *7*, respectively (Fig. 6). This suggests that they possess a self-regulatory and self-feedback mechanism. Therefore, the quick accumulation of their transcripts after exogenous ABA treatment may have connections with it.

At present, numerous reports are appearing about *ABF/AREB*s participating in dormancy. In *Arabidopsis*, *AtDPBF1/ABI5* was identified to be related to seed dormancy by regulating the *SOMNUS* gene (Chang et al., 2018; Lim et al., 2013) and flowering initiation (Wang et al., 2013). In sorghum, *SbABI5* was involved in grain dormancy (Rodríguez et al., 2009). Moreover, the expression of *TaABF1*, which is a homologous gene of *AtABI5*, was positively correlated with ABA sensitivity and seed dormancy in wheat (Rikiishi, Matsuura & Maekawa, 2010). A new study reported that in pear calli, *PpyABF3* participates in pear bud dormancy regulation by activating the expression of *PpyDAM3* (Yang et al., 2020). So, we speculate that *PmABFs* genes may also help regulate flower bud dormancy of *P. mume*.

## Conclusions

A total of 64 *ABF/AREB* genes were identified in nine Rosaceae species, which belonged to three traditional subfamilies: Rosoideae, Amygdaloideae, and Maloideae. The number of chromosomes (x) and *ABF/AREB* genes (n) varied with different subfamilies: Rosoideae (*x* = 7, *n* = 6), Amygdaloideae (*x* = 8, *n* = 7), Maloideae (*x* = 17, *n* = 9). Based on *AtABF/AREB*, the phylogenetic tree of these 64 *ABF/AREB* genes was built, indicating four subgroups (A, B, C, and D). In each group, the gene structures and the conserved motif compositions were similar. In *PmABF* gene promoters, there were three major types of *cis*-acting elements: light, hormones, and stresses response elements. All the *PmABFs* except *PmABF5* were sensitive to ABA. Several ABRE elements were contained in the promoters of *PmABF1*, *3*, *6*, *7*. Moreover, the expression levels of *PmABF3*, *4* and *6* were positively related to the ABA content during the dormancy stages. With the above in mind, we speculate that *PmABFs* may play a pivotal role in flower bud dormancy in *P. mume*.

##  Supplemental Information

10.7717/peerj.10785/supp-1 Supplemental Information 1 The ABF/AREB protein sequences of *Arabidopsis* and nine Rosaceae species

10.7717/peerj.10785/supp-2 Supplemental Information 2 The promoter sequences of *PmABFs*

10.7717/peerj.10785/supp-3 Supplemental Information 3 Genome information of the nine Rosaceae species

10.7717/peerj.10785/supp-4 Supplemental Information 4 Sequence information of the *ABF/AREB* subfamily members of nine Rosaceae species

10.7717/peerj.10785/supp-5 Supplemental Information 5 The primers for the qRT-PCR of *PmABFs*

10.7717/peerj.10785/supp-6 Supplemental Information 6 The Ka/Ks values of *ABF/AREB.* subfamily genes in *Prunus mume* vs. other eight Rosaceae species

10.7717/peerj.10785/supp-7 Supplemental Information 7 The *cis*-elements in the promoters of *PmABFs* genes

10.7717/peerj.10785/supp-8 Supplemental Information 8 Logos of the conserved motifs of *ABF/AREBs* sequences

10.7717/peerj.10785/supp-9 Supplemental Information 9 Chromosomal location of the *ABF/AREBs* in nine Rosaceae species A–I were *P. mume*, *P. dulcis*, *P. persica*, *P. armeniaca*, *M.* ×* domestica*, *P. betulifolia*, *R. Chinensis*, *R. occidentalis,* and *F. vesca*, respectively*.*

## Additional Information and Declarations

### Data Availability

The following information was supplied regarding data availability: Raw data, including protein sequences of ABF/AREBs in *Arabidopsis* and nine Rosaceae species and *PmABFs* promoter sequences, are available in the Supplemental File.
