# Supplementary material for: Genome-wide identification, characterisation, and evolution of ABF/AREB subfamily in nine Rosaceae species and expression analysis in mei (Prunus mume)
Source: PeerJ. 2021 Feb 4;9:e10785. doi: 10.7717/peerj.10785 (PMC7868070; doi:10.7717/peerj.10785)
Supplement: Supplemental Information 5 [file peerj-09-10785-s005.docx]

**Table S3**

**The primers for the qRT-PCR of *PmABFs***

| Name | Sequence |
| --- | --- |
| RT-PmABF1-F | GAATCGAGAGTCAGCAGCAA |
| RT-PmABF1-R | TCAGCTTTGTGTTCTCCTCTTTA |
| RT-PmABF2-F | GTGCTAGAAAGCAGGCATATCA |
| RT-PmABF2-R | TCCACCAAATCCTTCTCTTTCTT |
| RT-PmABF3-F | TTGGTCTGTTTCACTCAGATTT |
| RT-PmABF3-R | CTCGCTAATGGGTTGATTCC |
| RT-PmABF4-F | GACTGTTGAACGGAGACAGAA |
| RT-PmABF4-R | TCCTCCTCTAGACGTGAAACT |
| RT-PmABF5-F | AGCAGGCTTATACCAATGAGTT |
| RT-PmABF5-R | AGGCATGGAAGTAGGGTTTG |
| RT-PmABF6-F | AGTATTTCAAGGTGCCAGAAGA |
| RT-PmABF6-R | TGCCGGGCCAACATAAA |
| RT-PmABF7-F | CAATGCTGAAATGGCTACAAGG |
| RT-PmABF7-R | TTGGCGGCTAGGTTGTATTC |
| RT-PP2A-F | ATATAGCTGCTCAGTTCAACC |
| RT-PP2A-R | AAAAACAGTCACCACATTCTT |
