# Supplementary figures and images for: Genome-wide identification, characterisation, and evolution of ABF/AREB subfamily in nine Rosaceae species and expression analysis in mei (Prunus mume)
Source: PeerJ. 2021 Feb 4;9:e10785. doi: 10.7717/peerj.10785 (PMC7868070; doi:10.7717/peerj.10785)

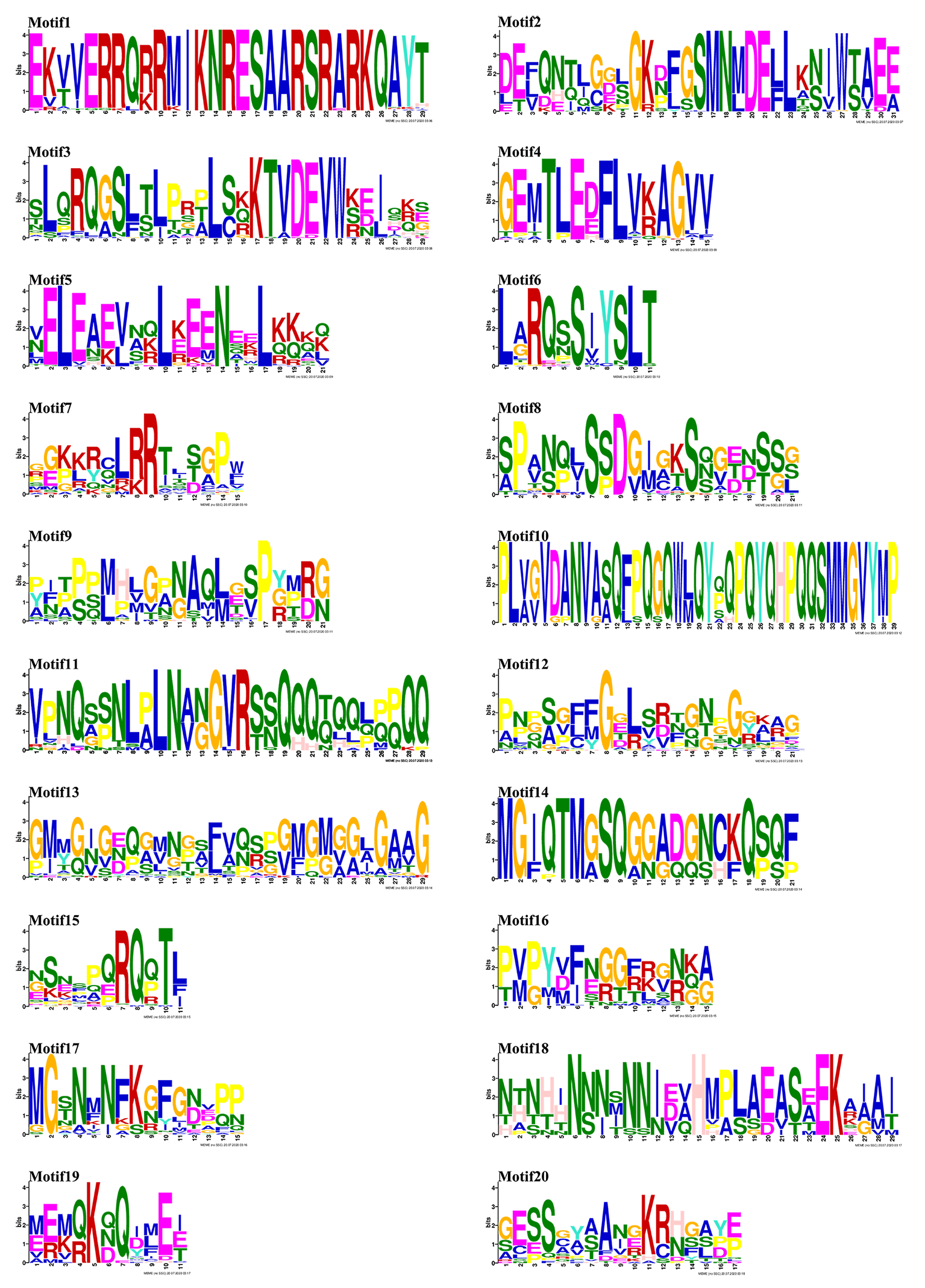

Supplement: Supplemental Information 8 [file peerj-09-10785-s008.png]

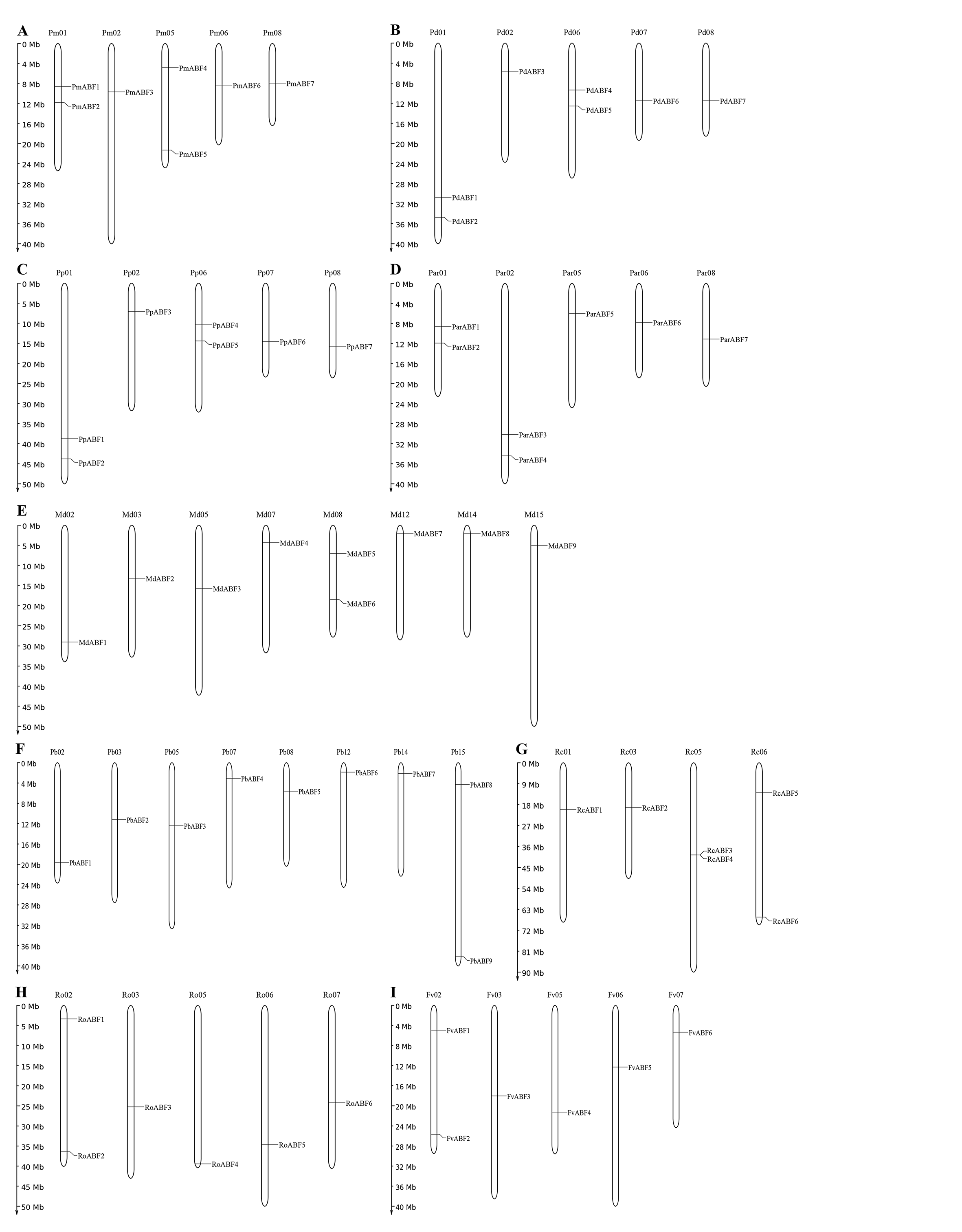

Supplement: Supplemental Information 9 — A–I were P. mume, P. dulcis, P. persica, P. armeniaca, M. × domestica, P. betulifolia, R. Chinensis, R. occidentalis, and F. vesca, respectively. [file peerj-09-10785-s009.png]
